# *Hevea brasiliensis* cell suspension peroxidase: purification, characterization and application for dye decolorization

**DOI:** 10.1186/2191-0855-3-14

**Published:** 2013-02-12

**Authors:** Thitikorn Chanwun, Nisaporn Muhamad, Nion Chirapongsatonkul, Nunta Churngchow

**Affiliations:** 1 Department of Biochemistry, Faculty of Science, Prince of Songkla University, Hat-Yai, Songkhla 90112, Thailand

**Keywords:** Cell suspension, Characterization, Decolorization, *Hevea brasiliensis*, Peroxidase, Purification

## Abstract

Peroxidases are oxidoreductase enzymes produced by most organisms. In this study, a peroxidase was purified from *Hevea brasiliensis* cell suspension by using anion exchange chromatography (DEAE-Sepharose), affinity chromatography (Con A-agarose) and preparative SDS-PAGE. The obtained enzyme appeared as a single band on SDS-PAGE with molecular mass of 70 kDa. Surprisingly, this purified peroxidase also had polyphenol oxidase activity. However, the biochemical characteristics were only studied in term of peroxidase because similar experiments in term of polyphenol oxidase have been reported in our pervious publication. The optimal pH of the purified peroxidase was 5.0 and its activity was retained at pH values between 5.0–10.0. The enzyme was heat stable over a wide range of temperatures (0–60°C), and less than 50% of its activity was lost at 70°C after incubation for 30 min. The enzyme was completely inhibited by β-mercaptoethanol and strongly inhibited by NaN_3_; in addition, its properties indicated that it was a heme containing glycoprotein. This peroxidase could decolorize many dyes; aniline blue, bromocresol purple, brilliant green, crystal violet, fuchsin, malachite green, methyl green, methyl violet and water blue. The stability against high temperature and extreme pH supported that the enzyme could be a potential peroxidase source for special industrial applications.

## Introduction

Peroxidases (EC 1.11.1.7) are enzymes that oxidize various hydrogen donors in the presence of H_2_O_2_ or organic hydroperoxides. They catalyse many different and important biochemical and physiological reactions in most living organisms. Plant peroxidases are involved in diverse physiological functions such as lignin biosynthesis (Gross 2008), suberization (Bernards et al. 1999), wound healing (Kumar et al. 2007), fruit ripening (Huang et al. 2007), auxin metabolism and disease resistance (Veitch 2004). The peroxidase superfamily can be divided into three classes according to their origin, amino acid homology and metal-binding capability. Class I includes the plant intracellular peroxidases as well as prokaryotic enzymes from mitochondria and chloroplasts (Passardi et al. 2007). Class II comprises extracellular fungal peroxidases: lignin-degrading peroxidase (LiP), and manganese peroxidase (MnP), and includes monomeric glycoproteins involved in the degradation of lignin. Fungal LiP and MnP belonging to this class of peroxidases have been most commonly studied for dye decolorization. Class III consists of secretory plant peroxidases, with multiple tissue specific functions; *e.g.* removal of H_2_O_2_ from chloroplasts and cytosol, oxidation of toxic compounds, biosynthesis of cell walls, defence responses towards wounding, indole-3-acetic acid catabolism, ethylene biosynthesis, etc. Some of the most well known peroxidases in this class are the horseradish peroxidase (HRP), turnip peroxidase (TP), bitter gourd peroxidase (BGP) and soybean peroxidase (SBP). Class III peroxidases are also monomeric glycoproteins containing four conserved disulphide bridges, and require calcium ions for their activities (Schuller et al. 1996). Peroxidases from plant tissues are able to oxidize a wide range of phenolic compounds, such as *o*-dianisidine, guaiacol, pyrogallol, chlorogenic acid, catechin and catechol (Passardi et al. 2005).

Peroxidases from different sources are of wide interest because of their extensive potential applications in the clinical, biochemical, biotechnological and industrial fields, and in the synthesis of useful compounds (e.g. various aromatic chemicals) (Srinivas et al. 2002). Because of their wide catalytic activities, these enzymes could be exploited for the detoxification and remediation of various aromatic pollutants; for example, phenols, aromatic amines, and dyes that have contaminated wastewater from the effluents of textile, printing, paper and pulp industries (Jadhav et al. 2009).

Peroxidases are found in vacuoles, tonoplasts, plasmalemma including inside and outside of the cell wall. These enzymes have been purified and studied from many plant sources; for example, oil palm (Deepa and Arumughan 2002), sweet potato tubers (Leon et al. 2002), melon (Rodriguez-Lopez et al. 2000), cauliflower (Koksal et al. 2008), rubber tree (Wititsuwannakul et al. 1997), baculovirus (Levin et al. 2005) and insect larvae (Loustau et al. 2008). In rubber trees (*Hevea brasiliensis* Willd. ex A. de Juss. Müell. Arg), peroxidase activity has been found in newly excised bark strips, possibly present in response to wounding (Wititsuwannakul et al. 1997).

In this report, a peroxidase was purified from cell suspensions of rubber tree using anion exchange chromatography, affinity chromatography and preparative SDS-PAGE. Unexpectedly, the purified peroxidase also possessed polyphenol oxidase activity inferring that it is a bifunctional enzyme. Since the properties of polyphenol oxidase was studied in the previous article (Muhamad et al. 2012), the purified enzyme was therefore analyzed based on the characteristics of peroxidase. The molecular weight of this peroxidase and some properties such as the effects of temperature, pH, some inhibitors and its activity on the dye decolorization were also reported. The obtained peroxidase may be used as an alternative source in some industrial applications since it was heat stable up to 70°C and its activity was retained over a wide range of pH values (5.0–10.0).

## Materials and methods

### *H. brasiliensis* cell suspension and culture condition

The cell suspension of *H. brasiliensis* was prepared according to Muhamad et al. (2012) and Te-chato et al. (2002). First, calli were cultivated from the integument of *H. brasiliensis* seeds on Murashige and Skoog’s (MS) medium containing 3% (w/v) sucrose, 1 mg/mL of 2,4-dichlorophenoxy acetic acid (2,4-D) and 1 mg/mL of 6-benzylaminopurine (BA), pH 5.7 under dark conditions at 25±2°C. The integument calli that developed were transferred to MS medium containing 2 mg/mL of 2, 4-D and 0.1 mg/mL of thidiazuron, pH 5.7 under controlled conditions for generating cell suspensions. The cells were subcultured to fresh medium every 14 days. The 28-day-old cell cultures were separated from the medium and stored at −20°C for the total protein extraction and further purification of peroxidase.

### Protein extraction, protein determination and peroxidase activity assay

Protein extraction was performed following some modifications of the procedure of Muhamad et al. (2012). Briefly, collected cells were ground with liquid N_2_ and extracted in 0.2 M phosphate buffer, pH 6.5, containing 0.25% (v/v) Triton X-100 and 3% (w/v) polyvinylpolypyrrolidone (PVPP). The supernatant was separated from the homogenate by centrifugation at 12,000 rpm and 4°C for 15 min. The protein content was measured by the Bradford method (Bradford 1976) using BSA as standard.

Peroxidase activity was assayed using a spectrophotometer according to (Shannon et al. 1996). The reaction mixture contained 2.775 mL of 0.05 M sodium acetate buffer, pH 5.4, 100 μL of 0.25% (w/v) *o*-dianisidine (ε = 11.3 mM^-1^cm^-1^ at 460 nm), 100 μL of 0.1 M H_2_O_2_ and 25 μL of enzyme solution. Absorbance at 460 nm was recorded every 15 s for 2 min. The enzyme activity was calculated following the formula below:

Enzyme activity (unit/mL) = $\frac{\mathrm{Vt}\times m}{\varepsilon \times D\times V}$ where *Vt* is the total volume of reaction (mL), *V* is the volume of enzyme (mL), *m* is the slope of the linear portion, *ε* is the molar extinction coefficient and *D* is the distance of the light pass which is 1 cm.

### Purification of peroxidase

The enzyme extract was subjected to a DEAE-Sepharose CL-6B column (2.5 cm x 5.0 cm, GE Healthcare) equilibrated with 0.02 M phosphate buffer, pH 8.0, at 4°C. Stepwise elution was done at a flow rate of 0.5 mL/min with the same buffer containing 0.05 and 0.1 M NaCl, respectively. The eluted fractions that exhibited high peroxidase activity were pooled and concentrated before loading onto a Con A-agarose column (1.5 cm × 1.5 cm, GE Healthcare) equilibrated with a solution containing 0.5 M NaCl, 0.005 M MgCl_2_, 0.005 M MnCl_2_ and 0.005 M CaCl_2_. The fractions with high peroxidase activity were eluted with 0.1 M methyl-α-mannopyranoside, concentrated and further purified by using preparative sodium dodecylsulfate-polyacrylamide gel electrophoresis (preparative SDS-PAGE). A single band of protein was extracted from the gel and dialyzed against 0.02 M phosphate buffer, pH 8.0.

### Tracking each step of peroxidase purification by electrophoretic analysis

The SDS- PAGE was carried out according to the method of Laemmli (1970). The molecular weight of the purified peroxidase was estimated based on the molecular weight markers (Bio-Sciences) after silver staining (GE Healthcare). The activity band was revealed by incubating in the mixture containing 1 mL of 0.25% (w/v) *o*-dianisidine, 1 mL of 0.1 M H_2_O_2_ and 28 mL of 0.05 M sodium acetate buffer, pH 5.4.

### Effect of temperature on enzyme stability

The purified peroxidase was incubated at various temperatures (0–80°C) for 30 min and then immediately put on ice. Enzyme activity was assayed as described above. The non-incubated enzyme was used as a control.

### Effect of pH on enzyme activity and stability

The optimum pH of the purified peroxidase was assayed by measuring its activity in standard buffer solutions of different pH values (pH 2.0–10.0) (Fluka). The pH stability was studied by incubating the enzyme with various buffer solutions (pH 2.0–10.0) for 30 min and then assayed for its activity after adjusting the pH to 5.4. The non-incubated enzyme was used as a control.

### Effect of various compounds on the peroxidase activity

The purified peroxidase was incubated with several compounds (EDTA, β-mercaptoethanol, NaN_3_, and SDS) at a ratio of 1:1 (v/v) for 5 min. The enzyme was incubated with distilled water for use as a control, and the enzyme activity was assayed as described above.

### The efficiency of the dye decolorization of the peroxidase

The dyes used in this study included amido black, aniline blue, brilliant green, bromocresol purple, bromothymol blue, congo red, crystal violet, erioglaucine, fast green, fuchsin, malachite green, metanil yellow, methylene green, methyl green, methyl orange, methyl red, methyl violet, resazurin, safranin T, toluidine blue O, trypan blue and water blue. The dye decolorization was assayed following the modified method of Parshetti et al. (2012). The reaction mixtures were prepared in distilled water. One mL of reaction mixture, that contains dye, 0.001 M of H_2_O_2_ and 25 units of the purified peroxidase, was incubated at room temperature and then the absorbance at the specific maximum wavelength of each dye was recorded at 24 h. The maximum wavelengths for the studied dyes were: amido black (620 nm), aniline blue (596 nm), brilliant green (626 nm), bromocresol purple (437 nm), bromothymol blue (434 nm), congo red (500 nm), crystal violet (590 nm), erioglaucine (628 nm), fast green (624 nm), fuchsin (543 nm), malachite green (617 nm), metanil yellow (444 nm), methylene green (664 nm), methyl green (632 nm), methyl orange (457 nm), methyl red (457 nm), methyl violet (584 nm), resazurin (600 nm), safranin T (521 nm), toluidine blue O (615 nm), trypan blue (595 nm) and water blue (596 nm). The decolorization was carried out with different starting amounts of each dye adjusted to provide the initial absorbance of 0.8–0.9 at its own specific wavelength. The percentage of the decolorization was calculated by the following formula;

%Decolorization = $\frac{\mathrm{ODu}-\mathrm{ODt}}{\mathrm{ODu}}$ × 100 where *ODu* is the absorbance of untreated while *ODt* is the absorbance of the treated dye at the desired incubation time. The decolorization process of each dye was observed in triplicate. The dye decolorization efficiency was analyzed by one-way ANOVA with the confidence level of 95%.

## Results

### Purification of peroxidase

**Figure 1:**
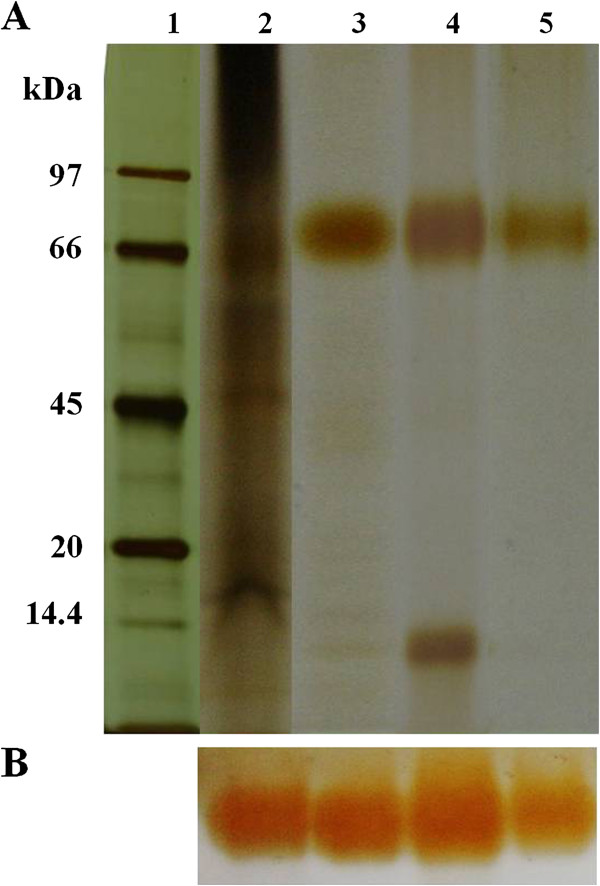
**The peroxidase from *H. brasiliensis *cell suspension, obtained from each purification step, on SDS-PAGE. (A)** The protein was revealed by silver staining. **(B)** The activity bands were revealed by staining with o-dianisidine. Lane1: molecular weight marker, lane 2: crude extract, lane 3: pooled fraction eluted by 0.1 M of NaCl on DEAE column, lane 4: pooled fraction eluted by 0.1 M of methyl-α-mannopyranoside on Con A-agarose column and lane 5: the eluted sample from the preparative SDS-PAGE.

**Figure 2:**
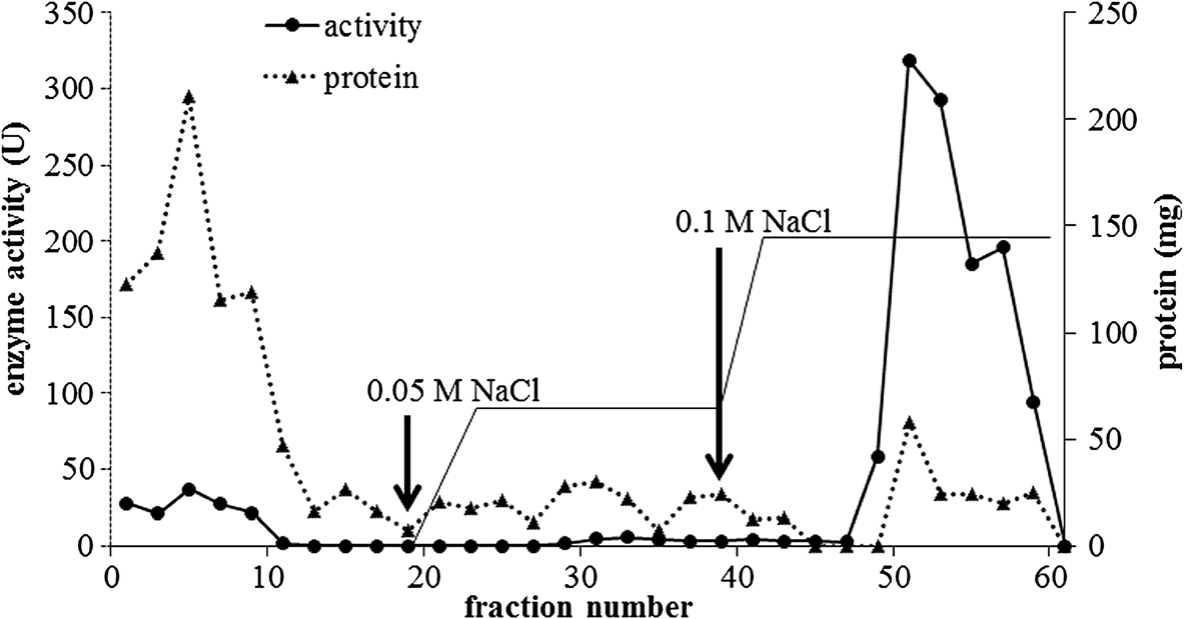
**Purification of the peroxidase enzyme from *H. brasiliensis *cell suspension by anion-exchange chromatography on DEAE-cellulose column and stepwise eluted by 0.05 M and 0.l M NaCl.** The arrows indicate the starting fraction that was eluted by each NaCl concentration. Each fraction was collected for 3 ml.

Several bands of peroxidase in the crude extract of *H. brasiliensis* cell suspension were detected after activity staining. The level of the major band increased with the age of the cell suspension (data not shown). The 28-day-old cell suspensions of *H. brasiliensis* were selected for the starting source for the peroxidase purification. Throughout this work, the peroxidase activity and activity staining (Figure 1B) was determined using *o*-dianisidine as the substrate. Three steps used for the purification included anion exchange chromatography (DEAE column), affinity chromatography (Con A-agarose column) followed by a preparative SDS-PAGE. From the DEAE column, fractions 50–60, eluted with 0.1 M NaCl, possessed the highest peroxidase activity (Figure 2). Those eluted fractions were pooled, concentrated and then transferred onto the Con A-agarose column. The fraction eluted with 0.1 M of methyl-α-mannopyranoside exhibited two bands on SDS-PAGE (Figure 1A). After further purification through the preparative SDS-PAGE, a single band that with a 44 fold increased in its specific activity was obtained (Figure 1A, Lane 5, Table 1). The purification steps, total activity, specific activity, purification factor and percentage of yield are summarized in Table 1. The molecular weight of the obtained peroxidase was 70 kDa according to the molecular weight markers on SDS-PAGE (Figure 1A).

**Table 1:** **Summary of the purification of the peroxidase isolated from a *H. brasiliensis *** **cell suspension**

**Purification step**	**Total protein (mg)**	**Total activity (U)**	**Specific activity (U/mg)**	**Purification factor**	**Yield (%)**
Crude	10.810	18796.46	1738.75	1	100.00
DEAE	0.213	797.95	3752.97	2.16	4.25
ConA-agarose	0.061	355.25	5820.53	3.35	1.89
Prep-SDS-PAGE	0.004	331.88	76872.67	44.21	1.77

### Optimum pH and effect of pH and temperature on the enzyme stability

**Figure 3:**
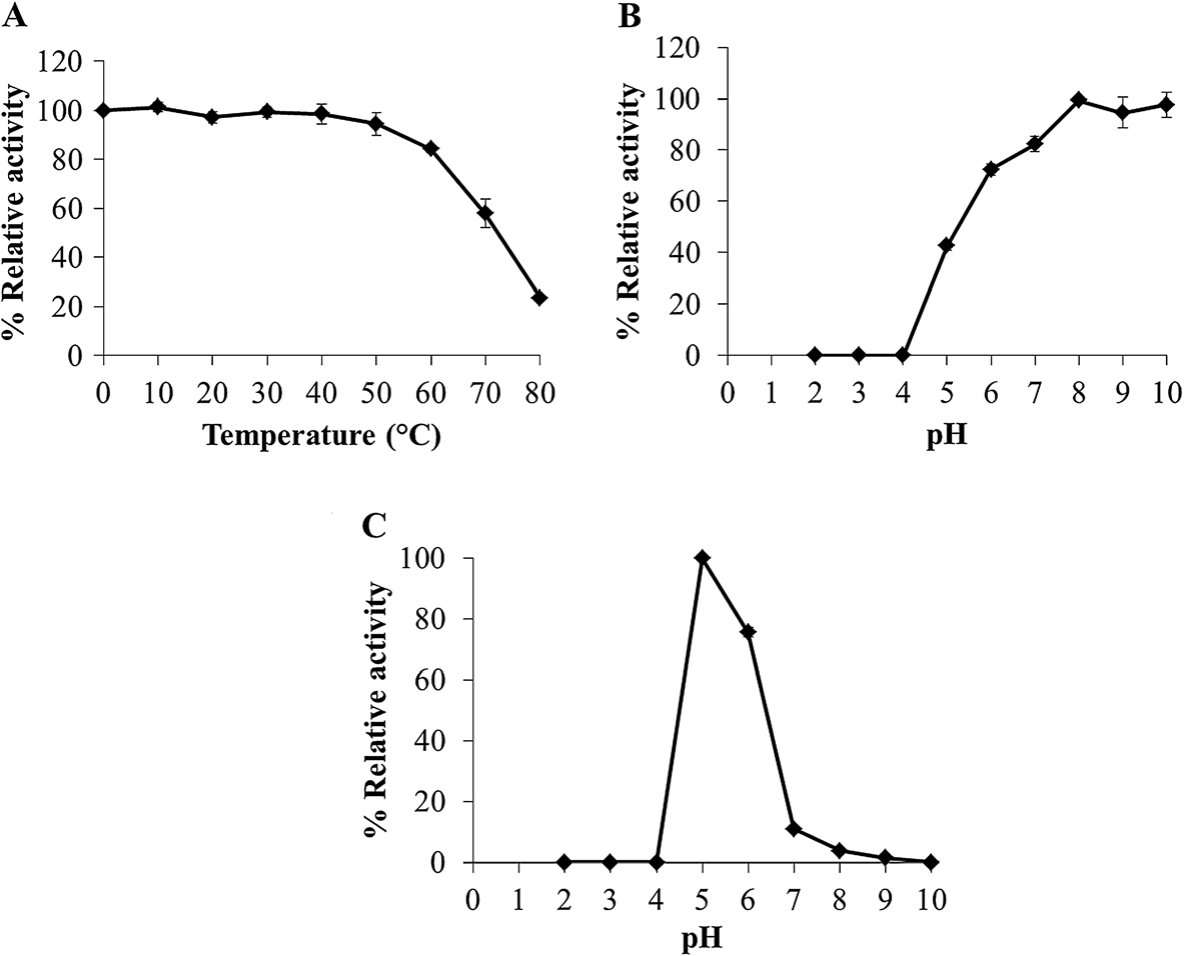
Effect of temperature and pH on the purified peroxidase: (A) temperature stability, (B) pH stability and (C) pH optimum.

The effect of pH on the activity of the purified peroxidase was determined over the range of pH between 2.0 and 10.0. The optimum pH of the purified enzyme was 5.0 (Figure 3C). For pH stability, the enzyme activity started to lose at pH below 7.0 and it was completely abolished at pH 2.0–4.0 (Figure 3B). The effect of temperature, 0–80°C, on the enzyme activity was also examined. The purified peroxidase retained 57% of its relative activity at 70°C (Figure 3A).

### Effect of various compounds on peroxidase activity

The effect of various compounds on peroxidase activity was determined at room temperature. The result showed that this enzyme was completely inhibited by β-mercaptoethanol and strongly inhibited by NaN_3,_ slightly inhibited by EDTA and SDS (Table 2).

**Table 2:** Effect of various compounds on the activity of the purified peroxidase, the results are shown as a mean of remained activity ± standard deviation of three replicates

**Compounds**	**Concentration**	**% Relative activity**
EDTA	1 mM	85.43±11.48
	5 mM	91.05±8.58
	10 mM	69.06±10.01
β-mercaptoethanol	1 mM	82.98±4.76
	5 mM	0.00
	10 mM	0.00
NaN_3_	1 mM	45.94±8.98
	5 mM	25.18±4.90
	10 mM	18.99±3.31
SDS	0.5%	84.75±5.20
	2.0%	86.51±4.69
	4.0%	89.16±4.83

### The efficiency of the peroxidase to catalyse dye decolorization

**Figure 4:**
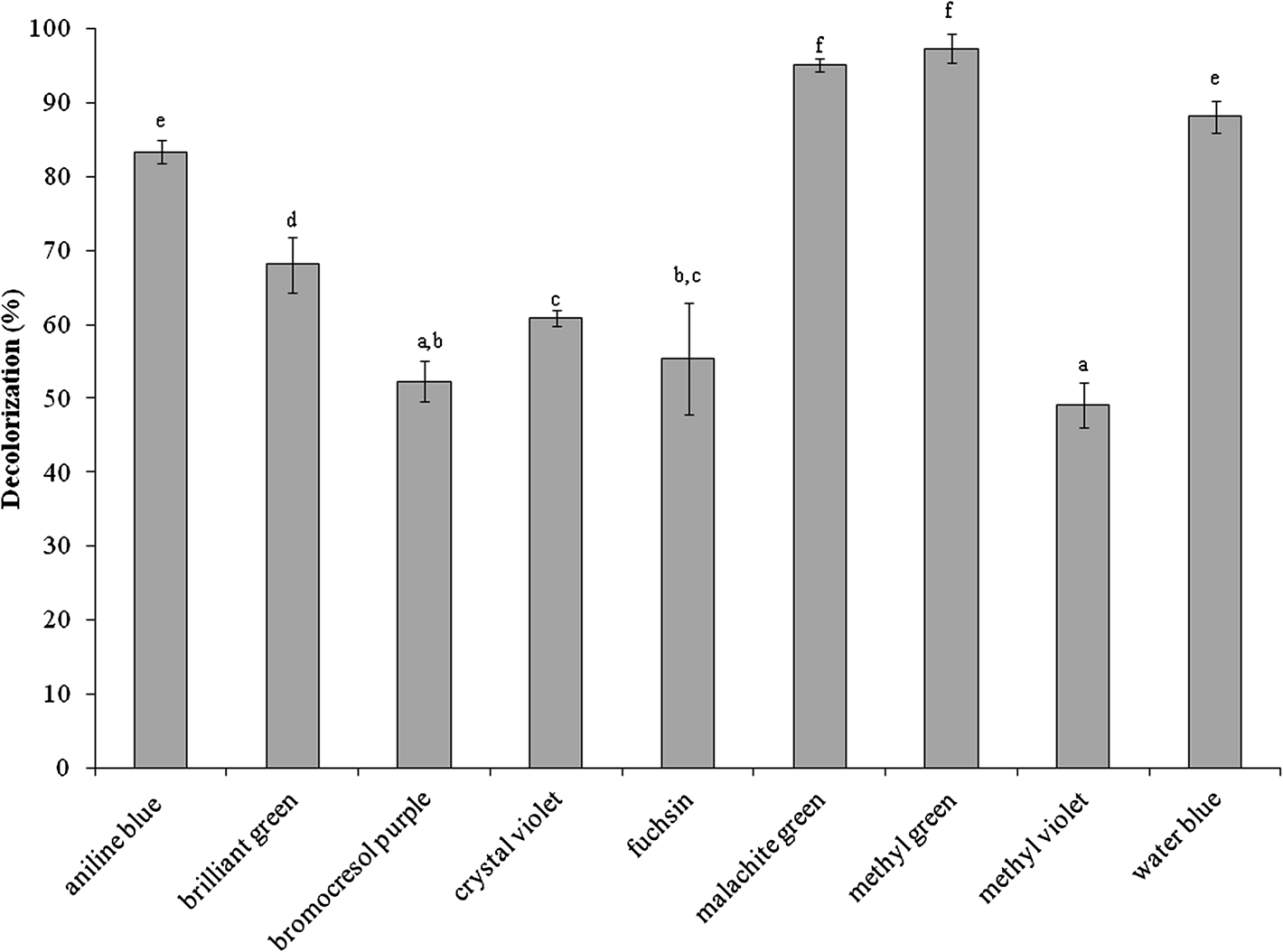
**The decolorization of tested dyes in triphenylmethane group.** Each bar represents mean ± standard deviation and letters a, b, c, d, e and f represent different level of significance of each dye (*p*<0.05).

The purified peroxidase (25 units) was tested for its ability to decolorize dyes that have been reported as contaminants in wastewaters from textile, plastic, paper industries and laboratory. The different groups of tested dyes included azo dye: methyl orange, methyl red and metanil yellow; diazo dye: amido black, congo red and trypan blue; oxazine dye: resazurin; phenazine dye: safranin T; phenothiazine dye: methylene green, toluidine blue O; and triphenylmethane dye: aniline blue, brilliant green, bromocresol purple, bromothymol blue, crystal violet, erioglaucine, fast green, fuchsin, malachite green, methyl green, methyl violet and water blue. Most of triphenylmethane dyes were decolorized while the other tested groups were not reacted (Table 3). Among these triphenylmethane dyes, various decolorization activities within 24 h were shown in Figure 4, aniline blue (83%), brilliant green (68%), bromocresol purple (52%), crystal violet (60%), fuchsin (55%), malachite green (95%), methyl green (97%), methyl violet (49%) and water blue (88%). However, all of the dyes in Figure 4 were decolorized to a clear solution after incubation for 72 h.

**Table 3:** Application of purified peroxidase on dye decolorization

**Groups**	**General chemical formula**	**Dyes**	**Decolorization effect**
Azo		Methyl orange	-
		Methyl red	-
		Metanil yellow	-
Diazo		Amido black	-
		Congo red	-
		Trypan blue	-
Oxazine		Resazurin	-
Phenazine		Safranin	-
Phenothiazine		Methylene green	-
		Toluidine blue O	-
Triphenylmethane		Aniline blue	+
		Brilliant green	+
		Bromocresol purple	+
		Bromothymol blue	-
		Crystal violet	+
		Erioglaucine	-
		Fast green	-
		Fuchsin	+
		Malachite green	+
		Methyl green	+
		Methyl violet	+
		Water blue	+

+ represent positive result of dye decolorization.

- represent negative result of dye decolorization.

## Discussion

A peroxidase enzyme from *H. brasiliensis* cell suspension was extracted, purified and characterized by determination of some of its biochemical properties and application in the dye decolorization. The results showed that the peroxidase was relatively easily purified through three steps, anion exchange chromatography (DEAE-Sepharose), affinity chromatography (Con A-agarose) then followed by preparative SDS-PAGE. The binding of the enzyme to the Con A-agarose column indicated that the peroxidase was a glycoprotein compatible with the previous studies reporting that most of the plant peroxidases were glycoproteins (Johansson et al. 1992; Kvaratskhelia et al. 1997; Passardi et al. 2005). Our purified peroxidase had a molecular weight of 70 kDa as determined by SDS-PAGE while the molecular weights of various peroxidases have been reported to be in the range of 30–150 kDa (Regalado et al. 2004). A similar molecular weight for the purified peroxidase has been previously reported from *Pseudomonas* sp. SUK1 (86 kDa), *Leptogium saturninum* (79 kDa) and *Kocuria rosea* MTCC 1532 (66 kDa), (Kalyani et al. 2011; Liers et al. 2011; Parshetti et al. 2012). The molecular weight of this obtained peroxidase was equal to those of the polyphenol oxidase previously characterized in our laboratory (Muhamad et al. 2012); however, at that time the peroxidase staining was not attempted. Now, the duplicated gels were stained with substrates of each enzyme and elucidated that it possessed the activities of both enzymes. By the mentioned purification procedure, the obtained protein also exhibited high activity of polyphenol oxidase inferring that it is a bifunctional enzyme. The bifunctional activity (peroxidase-polyphenol oxidase) also appeared in Satsuma mandarin, turnip and *Brassica oleracea* L. (Fujita et al. 1980a, 1980b ; Rahman et al. 2012). However, the fact that whether our purified protein is a single protein chain exhibiting both activities of peroxidase and polyphenol oxidase is being pursued to elucidate. In this report the enzyme was studied in term of peroxidase since our previous work has reported the biochemical characteristics of polyphenol oxidase enzyme (Muhamad et al. 2012). The optimal pH of the purified enzyme was 5.0 which were close to those reported from *Jatropha curcas*, *Streptomyces* sp. and *L. leucocephala* (Cai et al. 2012; Fodil et al. 2012; Pandey and Dwivedi 2011). For pH stability, its activity was retained at pH values between 5.0 and 10.0, and it had no activity at a pH < 4.0. Previous studies have reported that peroxidases lose their activities at low pH due to the instability of the heme molecule bound to the enzyme (Adams and Gorg 2002). Our purified peroxidase likewise contains heme groups since a peak of its spectrum was present at 302 nm (data not shown) which similar to the spectrum of other purified peroxidases containing heme groups (Gold et al. 1984; Kalyani et al. 2011; Tien and Kent Kirk et al. 1984). Moreover, the enzyme was heat stable over a wide range of temperatures (0–60°C), and about 40% of its activity was lost at 70°C within 30 min. The heat stability of this enzyme was similar to the peroxidases isolated from *Jatropha curcas*, artichoke and *Euphobia cotinifolia* (Cai et al. 2012; Cardinali et al. 2011; Kumar et al. 2011). The effect of various compounds on this peroxidase showed β-mercaptoethanol strongly inhibited its activity. This results indicated that at least one disulfide bond within the structure was important for its activity. Schuller et al. (1996) has reported that Class III peroxidases are monomeric glycoproteins; containing four conserved disulphide bridges. Our peroxidase was also the glycoprotein which contains disulphide bonds; therefore our enzyme may belong to class III peroxidase. The NaN_3_ could strongly inhibit this peroxidase. This chemical substance has been reported to be an inhibitor for all peroxidases (Pandey and Dwivedi 2011) as it can coordinate with the metal ion of a metal enzyme causing toxicity (Schwartz et al. 2001), for example, NaN_3_ acts as an inhibitor on a peroxidase from *Jatropha curcas* and *Viscum angulatum* (Cai et al. 2012; Das et al. 2011). Our purified enzyme was slightly inhibited by EDTA, a chelating agent, like those from *Jatropha curcas* and *Viscum angulatum* (Cai et al. 2012; Das et al. 2011). In addition, SDS which is a strong anionic detergent slightly inhibited its activity probably due to a conformational change of the enzyme. The enzymatic decolorization of dyes by this purified enzyme was examined using a UV–vis spectrophotometer. The results showed that the enzymatic activity could decolorize the triphenylmethane dye group. Aniline blue, malachite green, methyl green and water blue were rapidly decolorized (83–97%) within 24 h. This enzyme also decolorized brilliant green, bromocresol purple, crystal violet, fuchsin and methyl violet (49–68%) within 24 h, and the residuals were then cleared within 72 h. Recently, it has been reported that many aromatics dyes can be decolorized by peroxidase through the precipitation or breaking of the aromatic ring structure (Husain 2010). Many previous studies have also reported that bacterial and fungal peroxidases from, for example, *Phanerochaete chrysosporium* and *Bjerkandera adusta* could decolorize dyes (Bumpus and Brock 1988; Mohorčič et al. 2006). However using microorganisms to decolorize dyes involves high costs, alternative sources such as plants are now being considered. Our purified peroxidase could react on extreme condition, so it may be used as an alternative enzyme to treat water pollutants. Our study provided a new perspective for the use of this enzyme or a related system in environmental biotechnology. Further studies will focus on the purification and characterization of peroxidase enzymes from other tissues of *H. brasiliensis* such as leaves and on the identification of the products obtained from the decolorization process by this enzyme.

## Competing interests

The authors declare that they have no competing interests.

## References

[B1] Adams CD Gorg S Effect of pH and gas-phase ozone concentration on the decolorization of common textile dyes J Environ Eng 2002 3 293 298 10.1061/(ASCE)0733-9372(2002)128:3(293)

[B2] Bernards MA Fleming WD Llewellyn DB Priefer R Yang X Sabatino A Plourde GL Biochemical characterization of the suberization-associated anionic peroxidase of potato Plant Physiol 1999 3 135 146 10.1104/pp.121.1.135 10482668 PMC59361

[B3] Bradford MM A rapid and sensitive method for the quantitation of microgram quantitiesof protein utilizing the principle of protein dye binding Anal Biochem 1976 3 248 254 10.1016/0003-2697(76)90527-3 942051

[B4] Bumpus JA Brock BJ Biodegradation of crystal violet by the white rot fungus Phanerochaete chrysosporium Appl Environ Microbiol 1988 3 1143 1150 3389809 10.1128/aem.54.5.1143-1150.1988 PMC202618

[B5] Cai F Chao O Peipei D Shun G Ying X Fang C Purification and characterization of a novel thermal stable peroxidase from Jatropha curcas leaves J Mol Catal B Enzym 2012 3 59 66

[B6] Cardinali A Tursi N Ligorio A Giuffrida MG Napolitano L Caliandro R Sergio L Di Venere D Lattanzio V Sonnante G Purification, biochemical characterization and cloning of a new cationic peroxidase isoenzyme from artichoke Plant Physiol Biochem 2011 3 395 403 10.1016/j.plaphy.2011.01.028 21345687

[B7] Das MK Sharma RS Mishra V A novel cationic peroxidase (VanPrx) from a hemi-parasitic plant (Viscum angulatum) of Western Ghats (India): Purification, characterization and kinetic properties J Mol Catal B Enzym 2011 3 63 70 10.1016/j.molcatb.2011.03.010

[B8] Deepa SS Arumughan C Purification and characterization of soluble peroxidase from oil palm (Elaeis guineensis Jacq) leaf Phytochemistry 2002 3 503 511 10.1016/S0031-9422(02)00167-X 12409016

[B9] Fodil D Abdelmalek B Bassem J Nedia Z Fatma ZF Houcine B Purification and characterization of two extracellular peroxidases from Streptomyces sp. Strain AM2, a decolorizing actinomycetes responsible for the biodegradation of natural humic acids Int Biodeterior Biodegradation 2012 3 470 478

[B10] Fujita S Tono T Peroxidase activity of phloroglucin oloxidase from Satsuma mandarin fruits and effect of metal ions on the enzyme activity Nippon Nogeikagaku Kaishi 1980 3 201 208 10.1271/nogeikagaku1924.54.201

[B11] Fujita S Tono T Purification of phloroglucinoloxidase from turnip and its properties Nippon Nogeikagaku Kaishi 1980 3 429 435 10.1271/nogeikagaku1924.54.429

[B12] Gold MH Kuwahara M Chiu AA Glenn JK Purification and characterization of an extracellular hydrogen peroxide requiring diarylpropane oxygenase from the white rot basidiomycete, Phanerochaete chrysosporium Arch Biochem Biophys 1984 3 353 362 10.1016/0003-9861(84)90280-7 6497376

[B13] Gross GG From lignins to tannins: Forty years of enzyme studies on the biosynthesis of phenolic compounds Phytochemistry 2008 3 3018 3031 10.1016/j.phytochem.2007.04.031 17559893

[B14] Huang R Xia R Hu L Lu Y Wang M Antioxidant activity and oxygen-scavenging system in orange pulp during fruit ripening and maturation Sci Hortic 2007 3 166 172 10.1016/j.scienta.2007.03.010

[B15] Husain Q Peroxidase mediated decolorization and remediation of wastewater containing industrial dyes: a review Rev Environ Sci Biotechnol 2010 3 117 140 10.1007/s11157-009-9184-9

[B16] Jadhav UU Dawkar VV Telke AA Govindwar SP Decolorization of Direct Blue GLL with enhanced lignin peroxidase enzyme production in Comamonas sp UVS J Chem Technol Biotechnol 2009 3 126 132 10.1002/jctb.2017

[B17] Johansson A Rasmussen SK Harthill JE Welinder KG cDNA, amino acid and carbohydrate sequence of barley seed-specific peroxidase B P 1 Plant Mol Biol 1992 3 1151 1161 10.1007/BF00047718 1350932

[B18] Kalyani D Phugare S Shedbalkar U Jadhav J Purification and characterization of a bacterial peroxidase from the isolated strain Pseudomonas sp. SUK1 and its application for textile dye decolorization Ann Microbiol 2011 3 483 491 10.1007/s13213-010-0162-9

[B19] Koksal E Gulcin I Purification and characterization of peroxidase from cauliflower (Brassica oleracea L. var. botrytis) buds Protein Pept Lett 2008 3 320 326 10.2174/092986608784246506 18473941

[B20] Kumar S Dutta A Sinha AK Sen J Cloning, characterization andlocalization of a novel basicperoxidase gene from Catharanthus roseus FEBS J 2007 3 1290 1303 10.1111/j.1742-4658.2007.05677.x 17298442

[B21] Kumar R Singh KA Singh VK Jagannadham MV Biochemical characterization of a peroxidase isolated from Caribbean plant: Euphorbia cotinifolia Process Biochem 2011 3 1350 1357 10.1016/j.procbio.2011.03.003

[B22] Kvaratskhelia M Winkel C Thorneley RN Purification and characterization of a novel class III peroxidase isoenzyme from tea leaves Plant Physiol 1997 3 1237 1245 10.1104/pp.114.4.1237 9276947 PMC158416

[B23] Laemmli UK Cleavage of structural proteins during the assembly of the head of bacteriophage T4 Nature 1970 3 680 685 5432063 10.1038/227680a0

[B24] Leon JC Alpeeva IS Chubar TA Galaev IY Csoregi EI Sakharov IY Purification and substrate specificity of peroxidase from sweet potato tubers Plant Sci 2002 3 1011 1019

[B25] Levin G Mendive F Targovnik HM Cascone O Miranda MV Genetically engineered horseradish peroxidase for facilitated purification from baculovirus cultures by cation-exchange chromatography J Biotechnol 2005 3 363 369 10.1016/j.jbiotec.2005.05.015 16038999

[B26] Liers C Ullrich R Hofrichter M Minibayeva FV Beckett RP A heme peroxidase of the ascomyceteous lichen Leptogium saturninum oxidizes high-redox potential substrates Fungal Genet Biol 2011 3 1139 1145 10.1016/j.fgb.2011.10.004 22056522

[B27] Loustau MN Romero LV Levin GJ Magri ML López MG Taboga O Cascone O Miranda MV Expression and purification of horseradish peroxidase in insect larvae Process Biochem 2008 3 103 107 10.1016/j.procbio.2007.10.011

[B28] Mohorčič M Teodorovič S Golob V Friedrich J Fungal and enzymatic decolourisation of artificial textile dye baths Chemosphere 2006 3 1709 1717 10.1016/j.chemosphere.2005.09.063 16310823

[B29] Muhamad N Chirapongsatonkul N Churngchow N Defense-related polyphenol oxidase from Hevea brasiliensis cell suspension: Purification and characterization Appl Biochem Biotechnol 2012 3 177 189 10.1007/s12010-012-9690-z 22532343

[B30] Pandey VP Dwivedi UN Purification and characterization of peroxidase from Leucaena leucocephala, a tree legume J Mol Catal B Enzym 2011 3 168 173 10.1016/j.molcatb.2010.10.006

[B31] Parshetti G Parshetti S Kalyani D R-a D Govindwar S Industrial dye decolorizing lignin peroxidase from Kocuria rosea MTCC 1532 Ann Microbiol 2012 3 217 223 10.1007/s13213-011-0249-y

[B32] Passardi F Cosio C Penel C Dunand C Peroxidases have more functions than a Swiss army knife Plant Cell Rep 2005 3 255 265 10.1007/s00299-005-0972-6 15856234

[B33] Passardi F Bakalovic N Teixeira FK Margis-Pinheiro M Penel C Dunand C Prokaryotic origins of the non-animal peroxidase superfamily and organelle-mediated transmission to eukaryotes Genomics 2007 3 567 579 10.1016/j.ygeno.2007.01.006 17355904

[B34] Rahman ANF Ohta M Nakatani K Hayashi N Fujita S Purification and characterization of polyphenol oxidase from Cauliflower (Brassica oleracea L.) J Agric Food Chem 2012 3 3673 3678 10.1021/jf300380b 22471879

[B35] Regalado C García-Almendárez BE Duarte-Vázquez MA Biotechnological applications of peroxidases Phytochem Rev 2004 3 243 256

[B36] Rodriguez-Lopez JN Espin JC del Amor F Tudela J Martinez V Cerda A Garcia-Canovas F Purification and kinetic characterization of an anionic peroxidase from melon (Cucumis melo L.) cultivated under different salinity conditions J Agric Food Chem 2000 3 1537 1541 10.1021/jf9905774 10820055

[B37] Schuller DJ Ban N van Huystee R McPherson A Poulos TL The crystal structure of peanut peroxidase Structure 1996 3 311 321 10.1016/S0969-2126(96)00035-4 8805539

[B38] Schwartz B Olgin AK Klinman JP The role of copper in topa quinone biogenesis and catalysis, as probed by azide inhibition of a copper amine oxidase from yeast Biochemistry 2001 3 2954 2963 10.1021/bi0021378 11258907

[B39] Shannon ML Kay E Lew JY Peroxidase isozyme from horseradish root J Biochem 1996 3 2166 2172 5946638

[B40] Srinivas ND Barhate RS Raghavarao KSMS Aqueous two-phase extraction in combination with ultrafiltration for downstream processing of Ipomoea peroxidase J Food Eng 2002 3 1 6 10.1016/S0260-8774(01)00170-4

[B41] Te-chato S Hilae A Yeedum I Improve callus induction and embryogenic callus formation from cultured young leaves of oil palm seedling Thai J Agric Sci 2002 3 407 413

[B42] Tien M Kent Kirk T Lignin-degrading enzyme from Phanerochaete chrysosporium: Purification, characterization, and catalytic properties of a unique H2O requiring oxygenase Proc Natl Acad Sci USA 1984 3 2280 2284 10.1073/pnas.81.8.2280 16593451 PMC345042

[B43] Veitch NC Horseradish peroxidase: a modern view of a classic enzyme Phytochemistry 2004 3 249 259 10.1016/j.phytochem.2003.10.022 14751298

[B44] Wititsuwannakul R Wititsuwannakul D Sattaysevana B Pasitkul P Peroxidase from Hevea brasiliensis bark: purification and properties Phytochemistry 1997 3 237 241 10.1016/S0031-9422(96)00487-6

